# Spent coffee ground characterization, pelletization test and emissions assessment in the combustion process

**DOI:** 10.1038/s41598-021-84772-y

**Published:** 2021-03-04

**Authors:** A. Colantoni, E. Paris, L. Bianchini, S. Ferri, V. Marcantonio, M. Carnevale, A. Palma, V. Civitarese, F. Gallucci

**Affiliations:** 1 Department of Agriculture and Forestry Science (UNITUS-DAFNE), Tuscia University, Viterbo, Italy; 2 Centro Di Ricerca Ingegneria E Trasformazioni Agroalimentari (CREA-IT), Consiglio Per La Ricerca in Agricoltura E L’analisi Dell’economia Agraria (CREA), Rome, Italy

**Keywords:** Environmental sciences, Energy science and technology

## Abstract

Industrial development and increased energy requirements have led to high consumption of fossil fuels. Thus, environmental pollution has become a profound problem. Every year, a large amount of agro-industrial, municipal and forest residues are treated as waste, but they can be recovered and used to produce thermal and electrical energy through biological or thermochemical conversion processes. Among the main types of agro-industrial waste, soluble coffee residues represent a significant quantity all over the world. Silver skin and spent coffee grounds (SCG) are the main residues of the coffee industry. The many organic compounds contained in coffee residues suggest that their recovery and use could be very beneficial. Indeed, thanks to their composition, they can be used in the production of biodiesel, as a source of sugar, as a precursor for the creation of active carbon or as a sorbent for the removal of metals. After a careful evaluation of the possible uses of coffee grounds, the aim of this research was to show a broad characterization of coffee waste for energy purposes through physical and chemical analyses that highlight the most significant quality indexes, the interactions between them and the quantification of their importance. Results identify important tools for the qualification and quantification of the effects of coffee waste properties on energy production processes. They show that (SCG) are an excellent raw material as biomass, with excellent values in terms of calorific value and low ash content, allowing the production of 98% coffee pellets that are highly suitable for use in thermal conversion systems. Combustion tests were also carried out in an 80kW_th_ boiler and the resulting emissions without any type of abatement filter were characterized.

## Introduction

Day by day the massive usage of fossil fuels, due to the increasing energy demand, has led to a serious issue of environmental pollution. Every year, a large amount of agro-industrial, municipal, and forestry residues are treated as waste, while recent research indicates they can be recovered to produce thermal and electrical energy using biological or thermo-chemical conversion processes^1^. Identifying alternative energy sources is becoming increasingly important worldwide and their use has become essential in fulfilling the growing demand for energy, while at the same time trying to reduce possible environmental repercussions^2^. The European Union supports policies and strategies to develop greater awareness of sustainable attitudes and the use of renewable energy is one of the key targets^3,4^. Worldwide biomass ranks fourth as an energy resource, providing approximately 14% of the world's energy needs. Electricity production using renewable energy increased from 18.91% in 2008 to 25.60% in 2018, while CO_2_ emissions associated with worldwide electricity production increased from 28,605 MCO_2_ in 2008 to 32,915 MCO_2_ in 2018^5^. Among the main kinds of agro-industrial wastes, the residue of soluble coffee represents a significant amount worldwide. In fact, coffee is the second most traded commodity in the world after oil, and one of the most widely consumed beverages in the world^6^. Sustainable treatment and management solutions are needed to reduce the environmental impact of agro-food waste. These waste products produce odor and harborage for insects, which can increase environmental pollution if these by-products are not processed further^7^. Furthermore, the residues obtained from its production are proportionally increasing with growth in coffee consumption. Coffee silver skin and spent coffee grounds (SCG) are the most common coffee industry residues but also mucilage and parchment^8–10^. SCG residue obtained by combining raw coffee powder with hot water or steam for instant coffee preparation has fine particle size and high humidity (≈80 wt%), organic load and acidity. Moreover, this residue is produced in large amounts, with a worldwide annual generation of 6 million tons^11^. On average, one ton of green coffee generates about 650 kg of SCG and about 2 kg of wet SCG are generated for every single kg of soluble coffee produced^12^. As such, waste residue represents 50% of the input mass of coffee feedstock^13^. The many organic compounds contained in coffee residues suggest that their recovery and use could be very beneficial. Several researchers have studied coffee waste as a bio-resource for various valuable compounds. In the context of a circular economy, it is essential to re-use the many resources present in this residue, for example through fractionation, as demonstrated by Kovalcika et al. (2018)^14^; Caetano et al.^15^ evaluated the possibility of producing biodiesel from coffee waste; Mussatto et al.^8^ investigated coffee waste as a source of sugar; Kante et al.^16^ and Pappa et al.^17^ analyzed coffee waste as a precursor for creation of activated carbon and Oliveira et al.^18^ explored the possibility of using coffee waste as a sorbent for metal removal. The scope of the present work is to show an extensive characterization of coffee wastes for energy purposes through physical and chemical analyses that highlight the most significant quality indices, the interactions among them and the quantification of their importance. In addition, this research assesses SCG pelletizing at different blends of sawdust and gas and particulate emissions of the produced pellets. Note that the SCG have been collected and delivered by a coffee company without taking into account of the storage method which can have an effect on the energy yield of the biomass^19^. The residual biomass from the coffee that has been used in this work has been taken from the GEDAP company (located in Viterbo, in the center of Italy) and analyzed in the LASER-B laboratories of CREA—IT (located in Monterotondo, Rome).

## Materials and methods

### Biomass characterization

The SCG were provided by a local Viterbo company. The blend they use consists of 30% Arabica and the green beans are roasted between 200–230 degrees centigrade for about ten minutes. The characterization of SCG was investigated through chemical and physical analysis. The moisture content was determined in accordance with ISO 18134-1 (2015)^20^. The biomass sample was dried in a Memmert UFP800 oven at 105 ± 2 °C for 24 h. The moisture content was calculated by measuring the weight of the sample before and after drying. For biomass ashes analysis, the sample (1 g) was placed inside porcelain crucibles and then placed in the oven where it was first heated up to 250° C for 2 h with a ramp of 6.5 °C / min and then up to 550 °C for 1 h with a ramp of 10 °C / min, in accordance with ISO 18122. The higher heating value (HHV) was assessed using an Anton Paar 6400 isoperibol calorimeter. It was possible to obtain, by means of the value of the hydrogen content, lower heating value (LHV). The LHV was calculated using the relationship between HHV and LHV given by formula (1):

1 $$LHV = HHV - \left( {206*H/1000} \right)$$

where H equals the hydrogen percentages of the received tested fuels. The analysis of the higher heating value was made in accordance with ISO 18125.

The elemental composition in terms of carbon (C),hydrogen (H), nitrogen (N) and sulphur (S) was measured by means of the Costech ECS 4010 CHNS-O elemental analyzer and in accordance with EN 15104 (2011)^21^. Tin capsules, containing about 1 mg of the sample were prepared and inserted within the combustion furnace of the analyzer through a pneumatic carousel^22^.

The metal content was determinate by means of the ICP-MS 7700 Agilent in accordance with ISO 17294-2 (2016)^23^. To prepare the samples, an aliquot of each sample (about 500 mg) was transferred to special Teflon containers and subjected to acid attack using a microwave digester (Start D, Milestone, Italy). The etching mixture used was composed of 6 ml of HNO_3_ and 3 ml of H_2_O_2_. The determination of the metal content was carried out by ICP-MS (Inductively Coupled Plasma–Mass Spectrometer). All the solutions obtained were brought to a final volume of 100 ml with the addition of ultrapure water and subjected to analysis. The calibration of the instrument was carried out using multi-element standards (Standard mix, concentration 10 ppm in metal), prepared in aqueous solution acidified with 1% of HNO_3_. The calibration line was built using 5 standards in increasing concentration from 5 to 100 ppb. The Y was used as the internal standard through the automatic input system provided by the instrument^22^.

### Pellet

**Figure 1:**
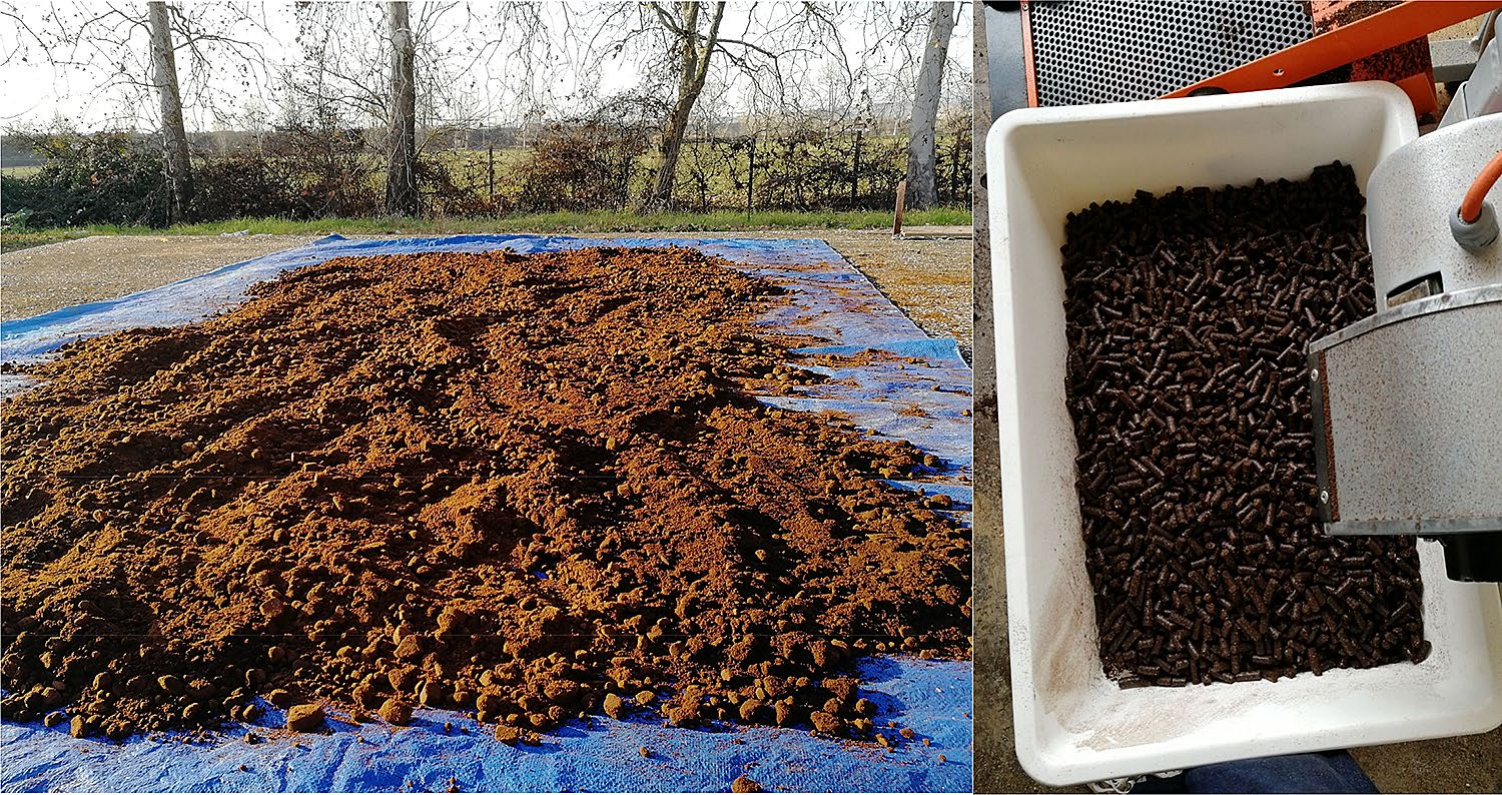
Spent coffee ground (SCG) before (left) and after (right) the pelletizing process.

Following the chemical-physical characterization of the coffee grounds, pelletizing tests were carried out using a Master 380 C Bianco-Line pellet machine and making various mixtures with sawdust, to check the best blend to use as the biomass (Fig. 1).The granular nature of the SGC requires an optimization of the mixture to be introduced into the pellet machine. The final product must have satisfactory structural properties without compromising the energy qualities of the starting matrix. The tests carried out were divided as follows:

1. 100% SCG;
2. 85% SCG and 15% sawdust;
3. 75% SCG and 25% sawdust;
4. 33% SCG and 66% sawdust.

The treatment without sawdust was obtained by mixing the SCG with 2% starch which was used as a binder. This percentage is within allowed limits of voluntary certification of the "ENplus" pellets.

Subsequently, the resulting pellets were analyzed for chemical-physical characteristics, as previously described for the SCG (elemental analysis, ash, higher and lower heating value).

### Emission

The study of the emissions produced by the combustion of the pellets made from SCG was conducted using a GSA / 80 boiler (D'Alessandro Termomeccanica GSA 80kWth Series). The boiler was equipped with a cast iron burner with a mechanical auger hearth and a cylindrical-shaped fuel loading hopper with a mechanical stirrer. The auger for the transport of fuel allows for adjustable speed which enables optimization of the biomass feeding. The auger also has an adjustable primary and secondary combustion air system. The stack is equipped with several flanges that allow the insertion of monitoring instruments and emission sampling devices. Table 1 shows the combustion parameters.

**Table 1:** Average combustion parameters.

Sampling and combustion values		
Velocity	1.33	[^m^/_sec_]
Stack temperature	161.91	[°C]
Stack pressure	101.34	[kPa]
Velocity at nozzle	1.311	[^m^/_sec_]
Probe temperature	114.4	[°C]
Filter temperature	155.6	[°C]
Outlet temperature	133.5	[°C]
Aux temperature	31.3	[°C]
Ambient pressure	101.35	[kPa]
Flow rate	0.0652	[^m3^/_sec_]
Biomass burning rate	21.22	[kg/h]

**Figure 2:**
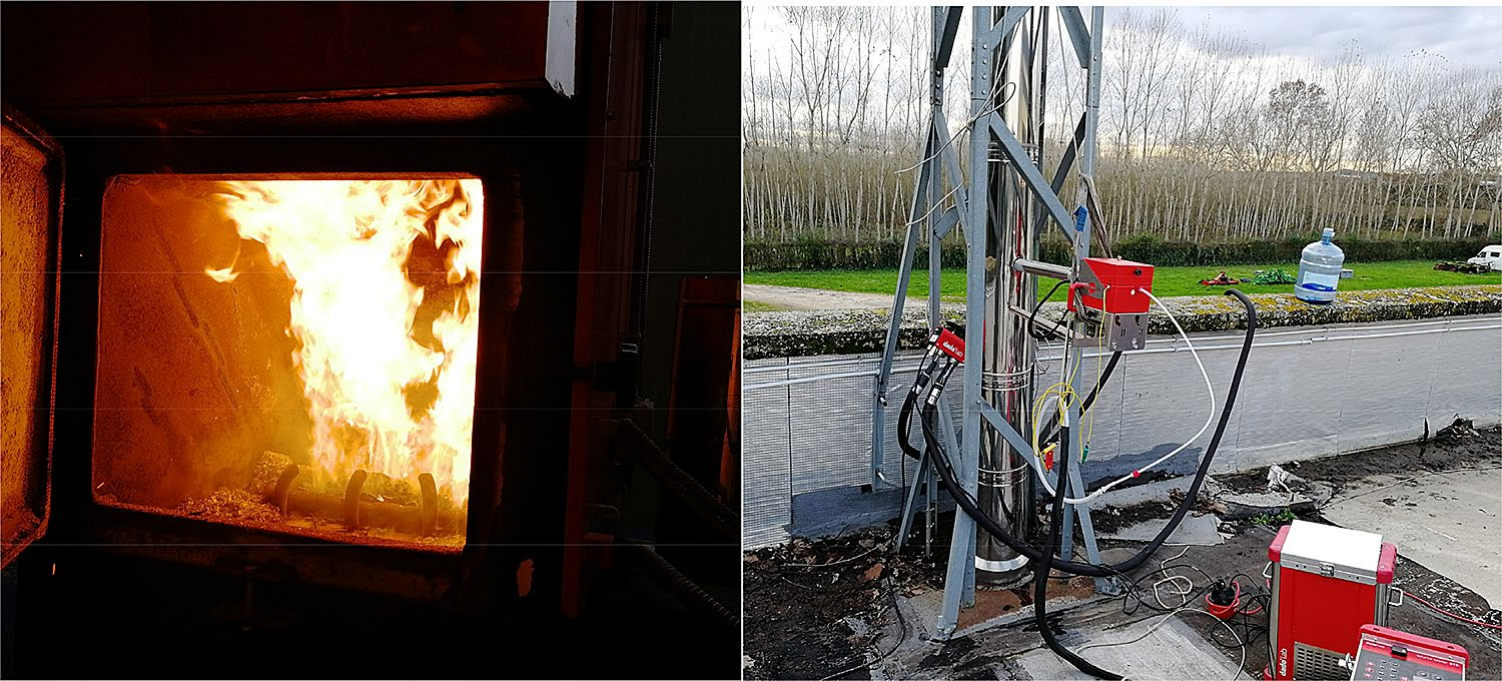
Combustion chamber (left) and probe system for sampling on the stack (right).

The online TOC analysis was conducted with a Ratfisch RS-53 T flame ionization detector (FID), compliant with the EN 12619 (2013)^24^ regulations, while the online analysis of NOx, CO, CO_2_, SO_2_ and O_2_ was carried out with a multiparametric detector Horiba PG-250, also in accordance with the main sampling methods and regulations. The instruments were calibrated before sampling with standard gas mixtures with defined concentration.Sampling of particulates, metals and VOCs were carried out in three different phases via the Dadolab HP5 heating probe interface with the stack. This probe is coupled to a pump Dadolab ST5 that allows sampling under isokinetic conditions (Fig. 2). The particulate matter was collected on a quartz fiber filter (ø 47 mm) suitably pretreated and preweighed as indicated by the European method EN 13284-1 (2017)^25^ and EPA 201A (2018)^26^. This filter was placed inside the heated chamber of the probe at 125 ± 5 °C and each sampling lasted 30 min.The metals following the ISO 14385 (2014)^27^ were sampled isokinetically with the same system, placing downstream of the quartz filter 3 bubblers maintained in a bath thermostated at 5 °C. The extremely volatile metals that manage to pass the quartz filter are thus trapped and condensed by the absorbent solutions contained in the bubblers. Each bubbler contained a solution of equal parts of HNO_3_ and H_2_0_2_ diluted in 9 parts of milliQ water. The third bubbler is defined as "control" and indicates that sampling is valid and meaningful only if the metals inside do not exceed 5% of those present in the 2 previous bubblers. The volatile organic compounds were sampled following the EN 13649 (2015)^28^ method by using self-made ACF (Activated Carbon Fibers) adsorbent traps suitable for subsequent thermal desorption. Each tube was interfaced with the probe via a swagelock fitting perpendicular to the emissive flow and a backup tube was placed at the end of it.Two volumes (40 ml and 160 ml) were sampled in triplicate with the aid of a suction pump located downstream of the system. The backup tube was used to verify that the volume breakthrough was not exceeded during sampling.

As part of the solution developed, the pretreated filters and the adsorbent tubes were kept aside and considered as analytical blanks. The PM (Total Suspended Particulate Matter TSP) was subsequently calculated by the quartz filter by gravimetry in relation to the volume of gas that passed through it. Metals were measured by ICP-MS by analyzing the quartz filter and the bubbled solutions. VOCs were measured with a thermal desorber (TD 100xr, Markes) coupled to a gascromatograph–mass spectrometer system (GC/MS–QQQ Agilent 7000).

The VOC’s analysis was performed with a 100-xr TD (Markes international) coupled to an Agilent 7000 GC/MS system. The tubes were thermo-desorbed with a flow of 50 ml / min up to a temperature of 350 °C for 10 min in splitless mode. They were collected on a focusing trap at the temperature of − 22 °C and then were redesorbed from the focusing trap for 1 min with a 1:10 split. The GC–MS analysis was carried out in splitless mode with following parameters specified in Table 2. The quantitative analysis was carried out by making calibration lines using a mixture of standards suitably produced by ULTRA Scientific Italia s.r.l. The qualitative analysis we resorted to the use of the Masshunter software interfaced with the NIST 08 library and the recognition was realized taking into consideration only the data with a match factor over 80%.

**Table 2:** VOC’s analysis operative parameters.

Operative parameters	
Carrier gas	He
Column	DB 502.2
Flow	1.2 ml/min
Mode (GC)	Constant Flow
Oven ramp	35 °C (5 min.) + 5 °C/min to 230 (5 min.)
Ion source	EI
Inlet	200 °C
MS source	230 °C
MSD transfer line	240 °C
Mode (MS)	Full scan 35–450 m/z

### Process modelling

The software Aspen Plus was chosen to model the experimental process. The following assumptions were made for the simulation:

- the process is steady state and isothermal^29^;
- drying and pyrolysis take place instantaneously and volatile products mainly consist of ${H}_{2, } CO, {CO}_{2}, {CH}_{4}$ and ${H}_{2}O$[25]^30^;
- char is 100% carbon^31^;
- all gases behave ideally.

**Figure 3:**
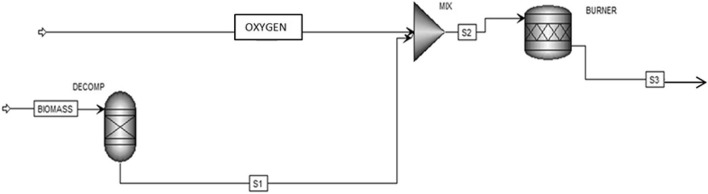
Flowchart of the plant evaluated in this study.

The process flow sheet is shown in Fig. 3.

The stream BIOMASS was specified as a non-conventional stream, as defined by its proximate and ultimate analysis. The BIOMASS stream goes to the RYELD reactor used to simulate the decomposition of the unconventional feed into its conventional components (carbon, hydrogen, oxygen, sulphur, nitrogen, and ash by the specification of the yield according to biomass ultimate analysis reported in Table 5). Off products from DECOMP moves into the mixer MIX in order to add the oxidising fluid, compose of pure air, to the combustible and then the resulting mixed S2 goes into the BURNER, to simulate the combustion process. The fumes out of the combustor are represented by stream S3.

Equations (1)–(4) listed in Table 3 are the chemical reactions evaluated in this work for the combustion process.

**Table 3:** Chemical reaction considered in the combustor^63^.

Reaction number	Reaction equation
1	C + 2H_2_ → CH_4_
2	C + O_2_ → CO_2_
3	C + 0.5O_2_ → CO
4	H_2_ + 0.5O_2_ → H_2_O
5	N_2_ + 3H_2_ → 2NH_3_

The following Table 4 shows the ASPEN units of the flowchart presented in Fig. 3.

**Table 4:** Description of ASPEN Plus flowsheet unit operation presented in Fig. 3.

ASPEN Plus name	Block ID		Description
RYIELD	DECOMP		Yield reactor—converts the non-conventional stream “BIOMASS” into its conventional components
MIXER	MIX		Mixer—mixes oxidising fluid with S1 stream that represents combustible fluid
RSTOIC	BURNER		Rstoic reactor—simulates the combustion process

### Statistical analysis

The characterization of biomass and pellet tests was performed using three samples as independent replicates. Data were analyzed using a one-factor analysis of variance (ANOVA) and a Pearson correlation test. Significance between means was determined using the Tukey post hoc multiple comparisons of means test at the 95% family-wise confidence level (*P* = 0.05). Before proceeding, normality and homogeneity of variance were checked via the Shapiro–Wilk test and Levene’s test, respectively (*P* = 0.05 for both of them). All statistical analyses were performed using JMP PRO 15 (Trial Version ©SAS Institute Inc.). The Pearson correlation and ANOVA with Tukey tests were applied to determine the relationship between elemental analysis, ash, and higher and lower heating value, first to characterize the SCG and second for pellet testing.

## Results

### Biomass characterization

The characterization allowed us to identify the features of biomass which are similar to the values reported in the literature (on average: C:68.52, H:11.04, N:1.40)^32^. SGS have a higher hydrogen content than other waste biomass such as olive, vine or hazelnut pruning (3.8%, 3.50%, 3.76% respectively); however, the amount of nitrogen is similar^33^. The moisture content plays an important role for the optimization of efficiency of thermal conversion processes. The samples as received had high moisture content of about 42% due to using water to prepare the drink. As a result, for all subsequent tests, SGS were dried, as described in materials and methods. As far as ash is concerned, SCG have shown excellent characteristics, with values not exceeding 1.3%, well below the range of 2–4% of common solid biomass. The lower heating value gave excellent results with values of about 20 MJ/kg and above on dry basis for the same biomass from timber, moreover the values showed stability in all samples tested (Table 5).

**Table 5:** Results of the SCG characterization and metals detected in SCG. Data are presented as mean ± standard deviation.

Samples	UM	SCG
Moisture	%	42.31 ± 6.85
Ash	%	0.90 ± 0.12
HHV	MJ/Kg	22.24 ± 0.05
LHV	MJ/Kg	19.97 ± 0.05
C	%	68.52 ± 10.20
H	%	11.04 ± 3.05
N	%	1.40 ± 1.20
S	%	< LoQ
Mg	mg/Kg	1981.71 ± 18.2
K	mg/Kg	1684.12 ± 7.14
Ca	mg/Kg	250.09 ± 4.3
Na	mg/Kg	37.15 ± 1.2
Al	mg/Kg	21.44 ± 3.6
B	mg/Kg	10.6 ± 4.5
Mn	mg/Kg	10.52 ± 2.6
Sr	mg/Kg	10.51 ± 2.01
Ga	mg/Kg	0.27 ± 0.3
Be	mg/Kg	< LoQ
Cr	mg/Kg	< LoQ
Co	mg/Kg	< LoQ
Ge	mg/Kg	< LoQ
As	mg/Kg	< LoQ
Ag	mg/Kg	< LoQ
Cd	mg/Kg	< LoQ
Fe	mg/Kg	9.87 ± 1.5
Cu	mg/Kg	9.82 ± 3.5
Zn	mg/Kg	3.57 ± 1.04
Ba	mg/Kg	3.17 ± 0.9
Ni	mg/Kg	1.23 ± 0.5
Li	mg/Kg	0.53 ± 0.5
In	mg/Kg	< LoQ
Sn	mg/Kg	< LoQ
Sb	mg/Kg	< LoQ
Cs	mg/Kg	< LoQ
Tl	mg/Kg	< LoQ
Pb	mg/Kg	< LoQ

The values of the elemental analysis were consistent with other biomass.

The analysis in ICP-MS allowed a characterization of the SGC matrix as regards to the metals present (Table 5). Large concentrations of macro elements are present, such as Mg, K, Ca and Na. In fact, besides being among the main characteristic metals of biomass, they are also the main mineral salts present in water and consequently their accumulation in the SGC is a consequence of making coffee. This is particularly important as it suggests that SGC coming from different areas with waters that have different chemical profiles will have different metal contents, as well as for all drinking water processes for instance ion exchangers for water softening. Otherwise, other metals have been detected in traces under the limit of quantification.

**Figure 4:**
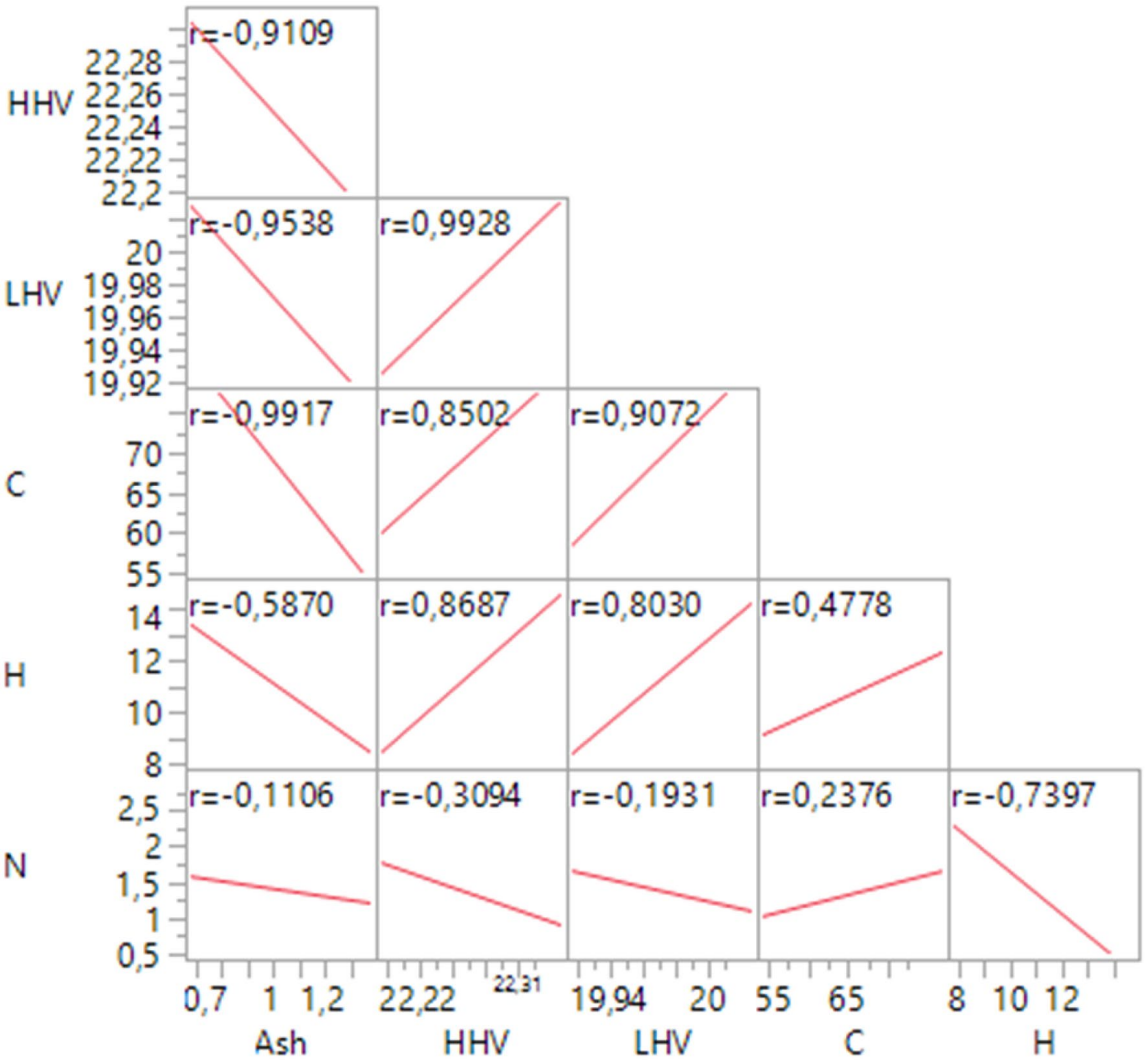
Matrix of Pearson correlation test of SCG characterization with 95% confidence interval.

According to the Pearson correlation test results, it was determined that there was a significant negative correlation between HHV, LHV and C with ash (P ≤ 0.05) and significant positive correlations between C and H with HHV and LHV (P ≤ 0.05) (Fig. 4). The correlation matrix provides interesting data indicating that heating values decrease with the increase of the ashes. This is because a higher percentage of ashes implies a greater quantity of unburnt products and consequently a lower calorific yield. Even nitrogen is involved in this effect as it is the only element whose increase in percentage corresponds to a decrease in heating value. This is partly due to the fact that a higher percentage of N corresponds to lower percentages of C, H and O (elements whose presence positively affects the increase in HHV and LHV); more significantly, the structure of nitrogen-rich biomasses contains more N atoms usually bonded with elements such as Si, Ca, K, Mn and others that are typically present in ashes. During combustion, nitrogen is emitted and all the elements bonded to it are found in ashes, increasing the percentage of the latter.

### Pellet

For the evaluation of the physico-chemical characteristics and heating values, four tests were performed mixing the coffee grounds with different percentages of sawdust (0%, 15%, 25%, 66%), to verify the presence of differences in the resulting pellets. The tests carried out with pellets made from SCG mixed with sawdust were analyzed in terms of heating value and elementary characterization. There were no sigificant differences in the carbon values of the treatments containing 0%, 15%, 25% and 66% of sawdust in the analysis of the variance ANOVA (*P* = 0.6602), although the treatments at the extremes (0% and 66%) resulted in greater percentage of carbon. The same was true for the hydrogen values including a higher presence of hydrogen in the treatment without sawdust. Significant differences were found in nitrogen and ash (Table 6) between the treatments with 66% sawdust and with no sawdust, while amounts of nitrogen and ash were similar for the treatments with 15% and 25% sawdust. As such, slightly higher values in ash and nitrogen were observed for the treatment using no sawdust.

**Table 6:** Elemental analysis and ashes of the 4 tests carried out. Data are presented as mean ± standard error. The letter in each row indicates a significant difference between the sawdust content at P ≤ 0.05.

% sawdust	Ash (%)	C (%)	H (%)	N (%)
0	2.19^a^ ± 0.27	51.32^a^ ± 0.41	3.09^a^ ± 0.19	2.97^a^ ± 0.01
15	1.91^ab^ ± 0.22	49.76^a^ ± 0.10	2.97^a^ ± 0.19	2.47^b^ ± 0.15
25	1.75^ab^ ± 0.19	50.40^a^ ± 0.16	2.74^a^ ± 0.18	2.27^b^ ± 0.01
66	1.19^b^ ± 0.21	51.48^a^ ± 0.29	2.62^a^ ± 0.06	1.75^c^ ± 0.12

**Figure 5:**
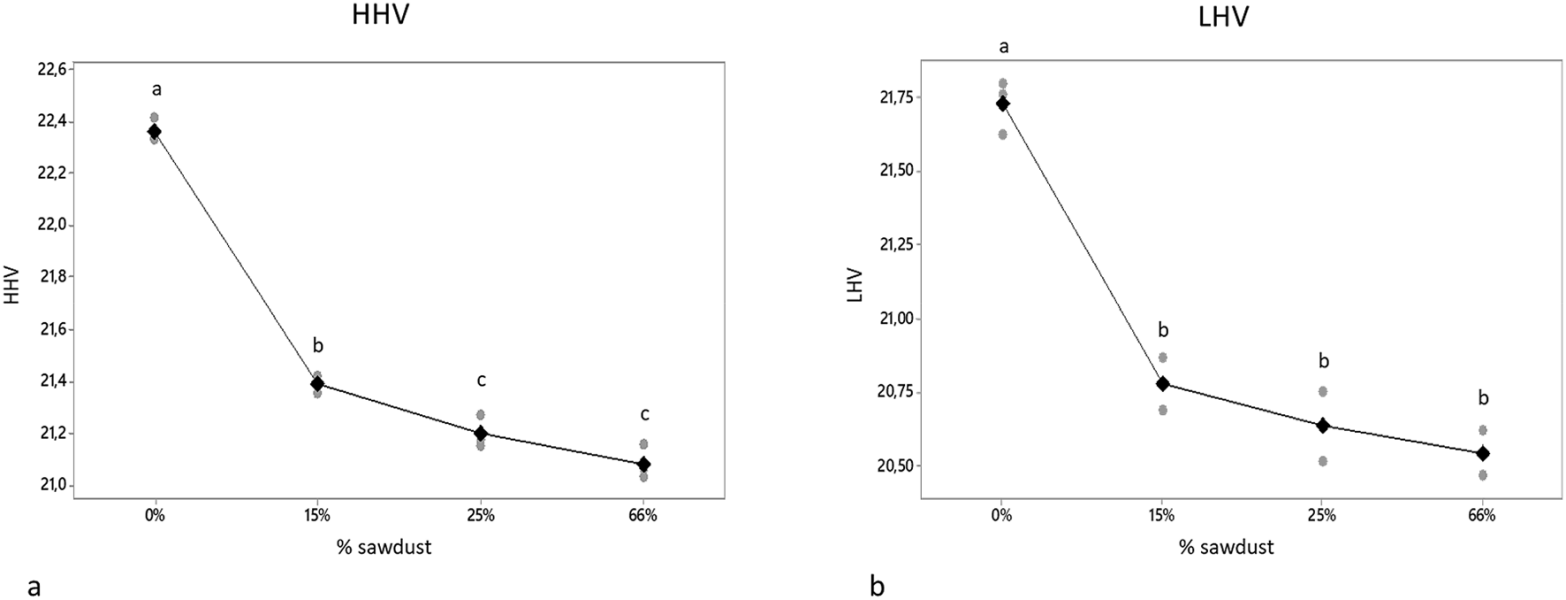
Higher and lower heating value of the pellet. The four types of pellets produced with the percentage of are indicated on the x axis sawdust. The letters indicate a significant difference between the sawdust content at P ≤ 0.05.

The pellets have an excellent level of heating value compare to other types of waste and to other biomasses, a fact that emerges from the comparison made by Zuorro and Lavecchia (2012) between SCG and other agricultural and forestry biomass or waste of which they report HHV, such as forest residues with 21.70 MJ/kg and a percentage variation of 3.04% with the HHV value of SCG obtained in this work, rice husks (15.29 MJ/kg, 46.24%), oak wood (18.70 MJ/kg, 19.57%), olive kernel (20.40 MJ/kg, 9.61%) and others^34^. The literature reports high levels of residual oil, which may be partially responsible for the high heating value^35^.The analysis showed decreasing values of HHV and LHV with increases in the percentage of sawdust. The best results were produced by the treatment without sawdust and the treatment consisting of 15% sawdust (Fig. 5), HHV values of 22.36 MJ/kg and 21.39 MJ/kg respectively. On the other hand, by increasing the percentage of sawdust in the pellets, a decrease in the amount of ash, a reduction in the amount of nitrogen and an increase in calorific value was observed. Hydrogen also showed a reduction; however, it was not statistically significant.

With regard to carbon, no differences were observed between the various pellets.

### Emission

After conducting the tests of the pellets obtained by mixing SGC and sawdust, it was clear that the addition of the latter, despite having an extraordinary amending effect, drastically lowered the extraordinary calorific value of the coffee (Fig. 5). Therefore, the water content of the starting matrix was optimized in order to eliminate sawdust and use water as a soil conditioner during the pelletizing phase. The optimization process led to the creation of pellets made of 90% dry SGC and 10% water, by weight (mean ± standard deviation 9.93 ± 0.51). The pellet produced was used in a boiler and the emissions produced by the combustion process were monitored.

#### Macropollutants

For atmospheric emissions, CO, NOx, O_2_, SO_2_, CO_2_ and carbon and TOC were evaluated. The hourly averages of emissions during the combustion process of 100% SCG are shown in Table 7. In addition, total particulate (TSP) was sampled and a value of 245.53 mg / Nm3 was found.

**Table 7:** Emission levels at different hours expressed in mg/Nm^3^.

Time h	CO_2_(%)	CO	NOx	SO_2_	TOC
1	9.22	3077.53	959.9	4.0	144.2
2	8.40	2129.25	1121.8	11.4	142.8
3	7.71	2153.74	1078.3	1.7	140.1

The obtained emission values are high for carbon monoxide, nitrogen oxides and powders. It is very important to specify that the simulated process is direct combustion without any abatement filter. It is likely that these values would be lower in common domestic boilers with abatement filters.

**Figure 6:**
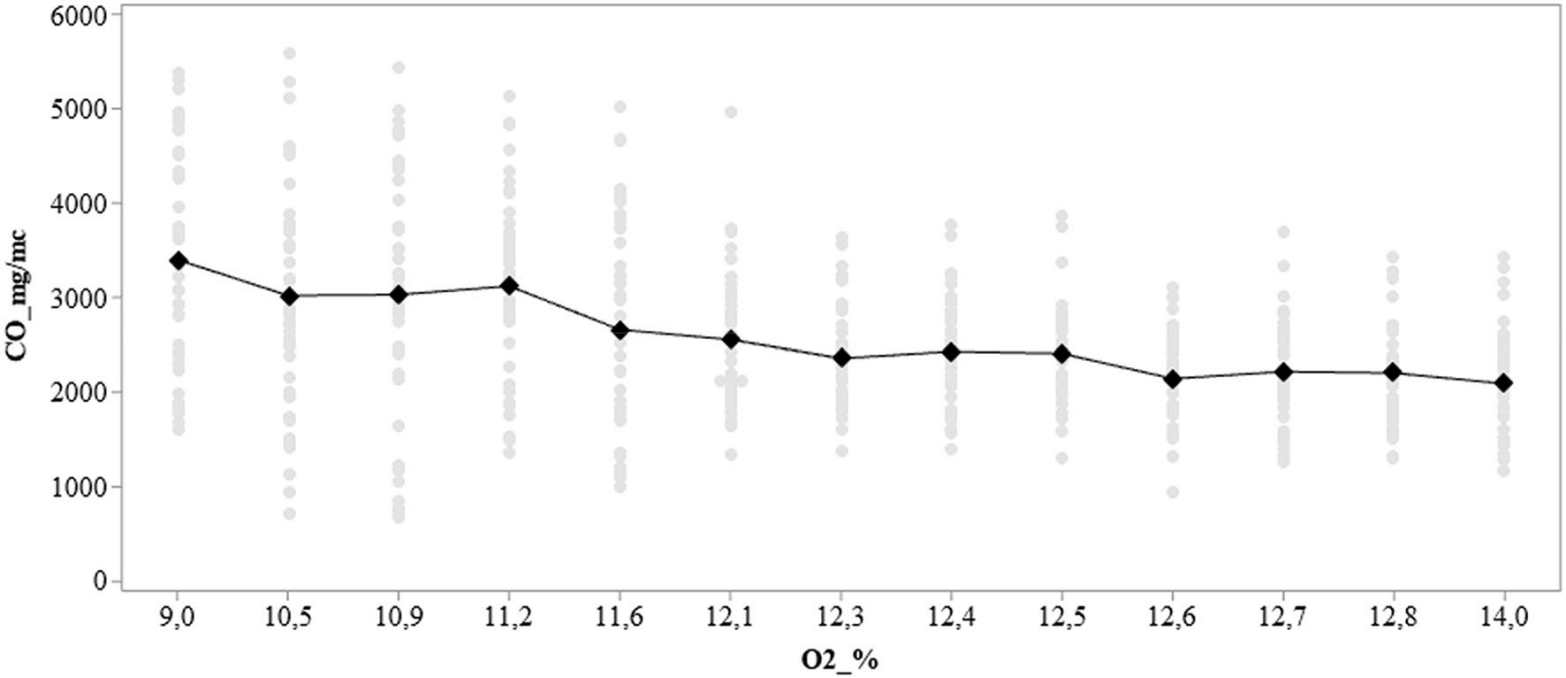
The decreasing CO trend is evident as the O_2_ content increases.The average CO trend tends to decline as the oxygen content increases.The oxygen levels represented on the X-axis reproduce the most frequent oxygen values found.

The Italian Legislative Decree 183/17 (which transposes Directive (UE) 2015/2193) establishes the limits for the emissions of boilers that use biomass by comparing pollutants to an oxygen content of 6% according to the formula (2):

2 $$C = \left[ {\left( {\left( {21 - O\_2 r} \right)} \right)/\left( {\left( {21 - O\_2 m} \right) } \right)} \right] \times Cm$$

where *O*_*2*_*r* is the percentage of reference oxygen content; *O*_*2*_*m* is the percentage of oxygen measured and *Cm* is the concentation of analyte measured. According to current legislation, there are no limits for CO2, CO and TOC for boilers with low thermal power (the lower limit is 0.15 MW). The only limit values indicated refer to NOx, SO2 and PM: 600 Mg/Nm^3^ NOx, 2000 Mg/Nm^3^ SO2, 200 Mg/Nm^3^ PM. However, if we take into consideration the values for low power boilers (0.15—5 MW), the CO limit is 525 mg / Nm3. NO_x_ and CO are above these limits (in particular CO is more than about 10 times the limit). CO was certainly the most important pollutant generated by the combustion process. The high concentration of these values ​​is probably due to the bad combustion conditions in terms of poor oxygen content during the combustion, with which it was operated. In fact, by graphing (Fig. 6) the trend of the CO as a function of the oxygen content, it can be observed that this significantly decreases with the increase of the comburent. In fact, a greater quantity of oxygen would allow compliance with stoichiometric ratios and a decrease in the formation of CO in favor of CO_2_, a zero-impact pollutant in the combustion of biomass, largely compensated by the sequestration of CO_2_ carried out by the growing biomass. Looking at the graph, it can be argued that by operating with greater quantities of oxidisers, it is already possible to significantly reduce pollutants, not to mention that the data shown was obtained without the use of abatement systems. As such, it is clearly possible to further decrease emissions.

#### VOCs

Forty-one main VOCs were identified by analyzing the tubes for thermal desorption. Usually the analysis of BTEX (Benzene, Toluene, Ethylbenzene, Xylenes) produces a highly indicative picture of VOC emissions (Table 8). These compounds are the most frequently monitored as they are the main volatile aromatic hydrocarbons which develop following all combustion processes. Of these, Benzene is certainly the most dangerous for humans and is designated a type 1 carcinogen by the IARC. We have chosen to quantify these compounds together with the other more prevelent compounds. The qualitative result of the profile of the generated VOCs is summarized in Appendix I.

The emission limit of benzene, in the Italian legislation relating to the emission limits for substances deemed carcinogenic and / or toxic for reproduction and / or mutagenic (in according to Italian Law n. 152/06 which transposes Directive (UE) 2015/2193), is equal to 5 mg/Nm^3^. In this case results are therefore well below the imposed limit.

#### Metals

Since the starting biomass is naturally low in metals except for those in the water used in the preparation process, the inorganic pollutants observed in the emissions have decidedly low concentrations (Table 9). It is important to note that heavy metals concentrations are very low and are even lower than LoQ.

Flyning ashes were decidedly poor in terms of metals, providing further proof that the matrix itself is quite free of metal contaminants. The problem of volatile and solid heavy metal compounds in emissions was analyzed to evaluate their behaviors. Results indicated that certain metals are less volatile and, for agricultural biomass, are concentrated in the ash.

In Table 10 is show the metal contenent in residual bottom ashes. The results underline that large parte of metals are concentred in this fraction.

**Table 8:** Main VOCs identified in the emissions. Bold the BTEXs and their classification for IARC.

Compounds	Concentration (mg/Nm^3^)	Emission factor (mg/kg fuel)	IARC Classification
2-Butene. 2-methyl-	4.99	55.17	–
Acetonitrile	1.51	16.69	–
Hexane	4.73	52.30	–
Benzene	3.74	41.35	1
Toluene	0.52	5.75	3
Ethylbenzene	0.26	2.87	2B
m.p-Xylene	0.31	3.43	3
o-Xylene	0.23	2.54	3
Benzene. 1.2.4-trimethyl-	3.16	34.94	–

### Combustion products obtained by Aspen Plus simulation

A model simulation using Aspen Plus software has been carried out in order to highlight the feasibility of the process. The level of CO2 in the emissions has been found to be around 10 mg/Nm^3^ while the level of NOx and SO2 are around 1000 mg/Nm^3^ and 10 mg/Nm^3^ respectively.

Considering a steady state simulation, with a biomass feed rate of 21.22 kg/h and an oxygen rate of 155 kg/h, the composition of the stream S3 is reported in Table 11. The process parameters of the burner are 700 °C and 1 bar.

In Table 11, the simulation results for the combustor outlet stream are compared with the results reported in Ref.^36^, achieved with air-combustion in a bubbling fluidised bed combustor. In Ref.^36^ the biomass considered is wood, since wood has similar properties to SCG, except for lower values of LHV, HHV, C%, N% and H% as showed in Ref^37^, this comparison makes sense and can be considered valid. and given the comparable features of SCG, the comparison can be considered valid. As shown in Table 11, the model results in more conservative values, perhaps due to the air staging method used in the experiment of ref.^36^, which is a well-known and effective method to control NOx emissions from solid fuel combustion boilers. Then, comparing the results of Table 11 with the results of Table 6, the values are consistent, showing the usefulness of the developed model.

## Discussion

Results identify important tools for the qualification and quantification of the effects of coffee waste properties on energy production processes.

The aim of the present study was to assess SCG pelletization with different blends of sawdust and gas, including the particulate emissions of the produced pellets (100% SCG).

Physico-chemical compositions of SCG, as well as the properties of the different pellets with different percentages of sawdust (0%, 15%, 25%, 66%), are presented in Tables 5 and 6 respectively. According to Table 6, the weight fractions of the different elements for SCG are similar to those present in the literature^38–40^.The main differences concern the ashes and nitrogen with average values of 0.90% and 1.40% respectively for SCG used in this study and 1.94% and 2.91% respectively from the study of Limousy et al*.* 2013^40^. The nitrogen content also varies widely in other studies; for example, Ktori^41^ and Liu^42^ found higher nitrogen values, respectively 3.51 and 2.48 (wt%), compared to 1.4 wt% in this study. This may be due to the influence of different geographical provenances and different coffee production systems. In fact, many studies have shown differences in the elementary composition of coffee based on geographical origin, as well as the type and quantity of metals present. Valentin and Watling (2013)^43^ have shown that, through elementary analysis, it is possible to distinguish the origins of coffee starting from the continent to the province, and in some cases, even to the plantation of origin, as verified by Habte et al*.* 2016^44^ in Ethiopia. Furthermore, their results show that these classifications are not affected by the roasting process, degree of ripeness or harvest date^43^. The results from this study showed that SCG have excellent values​​ in terms of heating value and low ash content, and this is also seen in other studies^40^. This is due to the increased concentration of sawdust which, regardless of species, has a lower calorific value than coffee grounds. Overall SCG presents as a typical biomass^45^ and has similar characteristics to both other coffee residues and other agro-industrial residues such as solid olive mill waste or grape vine^33,46,47^. In contrast, another coffee residue, mucilage, is different in its characteristics: (w/w) 85–91% water and between 6.2% and 7.4% sugars, with a high presence of nitrogen, It is used as fertilizer, feed or compost or for energy purposes in anaerobic digestion processes or for the production of ethanol, hydrogen and lactic acid^9,48^.

The presence of inorganic micropollutants that would proscribe the use of SCG as fuel was not found. However, the storage of biomass is an extremely important aspect, even more so if the purpose is energy use. **D**ifferent conditions and storage times on a matrix with a high level of humidity may or may not favour the proliferation of micro-organisms that may affect the physico-chemical characteristics and the energy yield^49^. Considering the high moisture content of SCG found, it is very important to act quickly for drying and storage and from what emerged during the trials, open air drying is not effective as a system due to the high hygroscopicity of the material, except at favourable latitudes and in the right season such as in the Mediterranean basin in the summer season, where temperatures and air humidity favour a stabilization of the biomass^19^. Whether or not to use inadequately stored biomass depends on the storage time, the climatic conditions, the composition and shape of the biomass, as well as the geometry and structure of the storage pile^49^.

Some properties obtained were compared with the limits of voluntary certification of the "ENplus" pellet. This certification system refers to the most important international regulations for pellet characteristics and makes them more stringent. Table 12 shows the limits of metals with the values obtained in this study. Some of these are well below the limits, while others—the ashes and nitrogen—are slightly higher.

**Table 9:** Concentration of metals in emissions and factor emission.

Metal	mg/Nm^3^	mg/kg fuel
B	18.20	201.22
Na	0.78	8.62
Mg	0.31	3.43
Al	0.78	8.62
K	0.55	6.08
Ca	0.75	8.3
Ti	0.35	3.87
Cr	0.02	0.22
Mn	0.01	0.11
Fe	0.23	2.54
Co	< LOQ	< LOQ
Ni	0.02	0.22
Cu	0.01	0.11
Zn	0.10	1.1
Ga	< LOQ	< LOQ
Se	0.45	4.98
Sr	< LOQ	< LOQ
Cd	< LOQ	< LOQ
Ba	< LOQ	< LOQ
Sc	0.22	2.43

**Table 10:** Bottom ashes express in mg/kg.

Na	Mg	Al	K	Ca	Cr	Mn	Fe
331.1892	4838.1773	184.2970	12,540.1292	4506.9221	11.3248	181.1106	645.6462
Ni	Cu	Zn	Ga	Sr	Ag	Cd	Ba
4.4608	42.9340	10.0459	1.3651	34.9875	0.0003	0.0080	26.2636
Tl	Pb	Bi					
0.0001	0.1105	0.0030

**Table 11:** Composition of the products of combustion.

	Model results	Literature data^38^
CO (mg/Nm^3^)	2679.23	3088.86
CO_2_ (mg/Nm^3^)	13.67	19.4
SO_2_ (mg/Nm^3^)	7.6	Not evaluated
NO_x_(mg/Nm^3^)	789.88	801.1

**Table 12:** Property of SCG pellet and limits of ENplus.

Property	Unit of measure	Limit	SCG
Moisture	%	≤ 10	9.93
Ash	%	≤ 2.0	2.19
LHV	MJ/kg	≥ 16.56	21.39
N	%	≤ 1.0	2.97
S	%	≤ 0.05	< LoQ
As	mg/kg	≤ 1	< LoQ
Cd	mg/kg	≤ 0.5	< LoQ
Cr	mg/kg	≤ 10	< LoQ
Cu	mg/kg	≤ 10	9.82
Pb	mg/kg	≤ 10	< LoQ
Ni	mg/kg	≤ 10	1.23
Zn	mg/kg	≤ 100	3.57
Hg	mg/kg	≤ 0.1	< LoQ

The pelletizing process is not easy to carry out, but the optimization of the process has made it possible to obtain pellets with a percentage of 90% of SCG with 2% starch, used as a binder. Furthermore, a significant reduction in the heating value was observed as the percentage of sawdust increases, as shown in Fig. 5, going from HHV of 22.42 MJ/kg to 21.03 respectively for 0% and 66% of sawdust.

The values ​​of higher heating value were compared with those of other biomasses and it emerged that with HHV of 22.24 MJ/kg, the SCG were found to have the highest value among those investigated^365241^.

Many studies have investigated the pellet of coffee grounds and some have found slightly lower heating values (18.8 MJ/kg^50^, 19.6 MJ/kg^51^) than that reported in this study while other report much higher heating values (23.4 MJ/kg^7^, 25.7 MJ/kg^52^). Other studies have verified the pelletizing process through mixtures with other types of waste^53^ or materials^54,55^, or with different percentages of moisture. Still others have modified other parameters such as die height, Lisowski et al. (2019)^56^ and have verified how the moisture of the matrix can be determined in the pelletizing process. In any case, it follows that coffee grounds have a great potential in energy conversion processes. In terms of chemical characteristics, the percentage of sawdust does not significantly affect the amount of C and H but affects nitrogen and ash which both decrease with increasing sawdust.

Some emission values​​ (PM, CO and NOx) were higher than regulatory limits, however it should be considered that the sampling was carried out in direct combustion without having any type of abatement system (multicyclone or bag filter).The presence of CO, however, rather than fuel, is attributable to the combustion conditions. It has been assumed (as shown in Fig. 6) that an increase in the oxidizer can improve the combustion conditions and lower the onset of CO.

Considering the relationship between metals content and emissions, the amount of alkali metals by pellets of various raw materials may be predicted based on fuel alkali metal content, K, and K + Na + Cl; both factors correlated with emissions. These findings highlight the importance of alkali metals with regard to PM1 emissions. Furthermore, CO decreased but there were higher NOX, SO2, and HCl emissions. In addition, fuel N content was found to correlate well with the NO_X_ emissions^45^.

By comparing the results of pellets from lignocellulosic and herbaceous biomass, the inorganic aerosol fractions are characterized by macro-pollutants as K, and high S concentrations in the emissions from combustion. Moreover, the contents of the easily volatile metals Zn, Pb, and Cd in emissions increased for wood fuels (standard softwood pellets, poplar). However, they can contain considerable amounts of transition metals, namely zinc^57–59^. In cases of just about complete combustion, zinc is the reason behind most of the cytotoxic studies^60–62^. Unlike agroforestry biomass, which results in emissions and ash with a higher content of metals, the use of SCG is advantageous due to its low metal concentration in both emissions and ash. This allows better management of the residual ash from the combustion of SCG which can be used as is, unlike the ash derived from biomass, which requires further treatment before disposal.

## Conclusions

For this study, SCGs of a local Viterbo (Italy) company was used. The physical–chemical characteristics of the SCGs confirmed the very high quality of the raw material as a biomass, much higher than that for wood biomass, showing excellent levels of calorific value (HHV 22.36 MJ/kg) and negligible values of polluting metals. The dusty nature of SCG causes some difficulties in the pelletizing phase. This obstacle can be easily overcome by mixing the SCG matrix with sawdust; however, results indicate that this practice significantly reduces the energy qualities of the SCG. The best alternative was therefore to use a mixture of wet and dry coffee in order to establish the optimum moisture content for pelletization.

High values were evident for some of the atmospheric emissions, but it is important to point out that the process used was direct combustion without any abatement filter. It is likely that these values would be lower when using common domestic boilers with abatement filters.

SPG have excellent values in terms of calorific value and low ash content. The optimization of the process allowed the production of pellets containing 90% SCG. The results indicate that SCG an excellent candidate for use in energy conversion systems.

## Supplementary Information

Supplementary Information 1.

Supplementary Information 2.

Supplementary Information 3.
