# Supplementary material for: Spent coffee ground characterization, pelletization test and emissions assessment in the combustion process
Source: Sci Rep. 2021 Mar 4;11:5119. doi: 10.1038/s41598-021-84772-y (PMC7933292; doi:10.1038/s41598-021-84772-y)
Supplement: Supplementary file 2 — Supplementary Information 2. [file 41598_2021_84772_MOESM2_ESM.docx]

Spent coffee ground characterization, pelletization test and emissions assessment in the combustion process

Colantoni, A.^1*^,Paris, E.^2^,Bianchini, L.^1*^,Ferri, S.^1^, Marcantonio, V.^1^, Carnevale, M.^1^, Palma, A.^2^, Civitarese, V.^2^,Gallucci, F.^2^

^1^ Tuscia University – Department of Agriculture and Forestry Science (UNITUS-DAFNE); *l.bianchini@unitus.it

^2^ Consiglio per la ricerca in agricoltura e l’analisi dell’economia agraria (CREA) – Centro di ricerca Ingegneria e Trasformazioni agroalimentari (CREA-IT);

**Highlights**

- A study was conducted on the re-use of coffee grounds as an energy source.
- Spent coffee grounds can be employed as pellet to obtain high-value products.
- The biomass combustion products have been evaluated experimentally.
- Simulative combustion model was developed with Aspen Plus.

***Corresponding author:** [**colantoni@unitus.it**](mailto:colantoni@unitus.it)**, Tuscia University, +39 0761357356**

**Abstract**

Industrial development and increased energy requirements have led to high consumption of fossil fuels. Thus, environmental pollution has become a profound problem. Every year, a large amount of agro-industrial, municipal and forest residues are treated as waste, but they can be recovered and used to produce thermal and electrical energy through biological or thermochemical conversion processes. Among the main types of agro-industrial waste, soluble coffee residues represent a significant quantity all over the world. Silver coffee peel and spent coffee grounds are the main residues of the coffee industry. The many organic compounds contained in coffee residues motivate their enhancement. Thanks to its composition it can be used in the production of biodiesel, as a source of sugar, as a precursor for the creation of active carbon or it can be used as a sorbent for the removal of metals. After a careful evaluation of the possible use of coffee grounds, the aim of this work was to show a broad characterization of coffee waste for energy purposes, through physical and chemical analyses that highlighted the most significant quality indexes, the interactions between them and the quantification of their importance. This study provides results that are important tools for the qualification and quantification of the effects of coffee waste properties on energy production processes. It shows that SCGs are an excellent raw material as biomass, showing excellent values in terms of calorific value and low ash content that have allowed the obtainment of 98% coffee pellets excellent for use in thermal conversion systems.Combustion tests were also carried out in an 80kW_th_ boiler and the emissions produced, without any type of abatement filter, were characterized.

**Highlights**

- A study was conducted on the re-use of coffee grounds as an energy source.
- Spent coffee grounds can be employed as pellet to obtain high-value products.
- The biomass combustion products have been evaluated experimentally.
- Simulative combustion model was developed with Aspen Plus.

***Corresponding author:** [**colantoni@unitus.it**](mailto:colantoni@unitus.it)**, Tuscia University, +39 0761357356**
