# Supplementary material for: Spent coffee ground characterization, pelletization test and emissions assessment in the combustion process
Source: Sci Rep. 2021 Mar 4;11:5119. doi: 10.1038/s41598-021-84772-y (PMC7933292; doi:10.1038/s41598-021-84772-y)
Supplement: Supplementary file 3 — Supplementary Information 3. [file 41598_2021_84772_MOESM3_ESM.docx]

Spent coffee ground characterization, pelletization test and emissions assessment in the combustion process

Colantoni, A.^1*^,Paris, E.^2^,Bianchini, L.^1*^,Ferri, S.^1^, Marcantonio, V.^1^, Carnevale, M.^1^, Palma, A.^2^, Civitarese, V.^2^,Gallucci, F.^2^

^1^ Tuscia University – Department of Agriculture and Forestry Science (UNITUS-DAFNE); *l.bianchini@unitus.it

^2^ Consiglio per la ricerca in agricoltura e l’analisi dell’economia agraria (CREA) – Centro di ricerca Ingegneria e Trasformazioni agroalimentari (CREA-IT);

**Highlights**

- A study was conducted on the re-use of coffee grounds as an energy source.
- Spent coffee grounds can be employed as pellet to obtain high-value products.
- The biomass combustion products have been evaluated experimentally.
- Simulative combustion model was developed with Aspen Plus.

***Corresponding author:** [**colantoni@unitus.it**](mailto:colantoni@unitus.it)**, Tuscia University, +39 0761357356**

**Abstract**

Industrial development and increased energy requirements have led to high consumption of fossil fuels. Thus, environmental pollution has become a profound problem. Every year, a large amount of agro-industrial, municipal and forest residues are treated as waste, but they can be recovered and used to produce thermal and electrical energy through biological or thermochemical conversion processes. Among the main types of agro-industrial waste, soluble coffee residues represent a significant quantity all over the world. Silver coffee peel and spent coffee grounds are the main residues of the coffee industry. The many organic compounds contained in coffee residues motivate their enhancement. Thanks to its composition it can be used in the production of biodiesel, as a source of sugar, as a precursor for the creation of active carbon or it can be used as a sorbent for the removal of metals. After a careful evaluation of the possible use of coffee grounds, the aim of this work was to show a broad characterization of coffee waste for energy purposes, through physical and chemical analyses that highlighted the most significant quality indexes, the interactions between them and the quantification of their importance. This study provides results that are important tools for the qualification and quantification of the effects of coffee waste properties on energy production processes. It shows that SCGs are an excellent raw material as biomass, showing excellent values in terms of calorific value and low ash content that have allowed the obtainment of 98% coffee pellets excellent for use in thermal conversion systems.Combustion tests were also carried out in an 80kW_th_ boiler and the emissions produced, without any type of abatement filter, were characterized.

**List of abbreviations**

SCG Spent coffee grounds

HHV Higher Heating Value

LHV Lower Heating Value

ACF Activated Carbon Fibers

FID Flame Ionization Detector

BTEX Benzene Toluene Ethylbenzene Xylenes

TOC Total Organic Carbon

VOCs Volatile OrganicCompounds

PM Particulate Matter

ANOVA Analysis Of Variance

% Percentage

°C Degrees Celsius

kg Kilogram

ml Millilitre

ppm Part per million

ppb Parts per billion

MJ/kg Megajoules per kilogram

mg/Nm3 Milligrams per normal cubic metre

mg/kg Milligrams per kilogram

LoQ Limit Of Quantification

Ag Silver

Al Aluminium

As Arsenic

B Boron

Ba Barium

Be Beryllium

C Carbon

Ca Calcium

Cd Cadmium

Cl Chlorine

Co Cobalt

Cr Chromium

Cs Caesium

Cu Copper

Fe Iron

Ga Gallium

Ge Germanium

H Hydrogen

In Indium

K Potassium

Li Lithium

Mg Magnesium

Mn Manganese

N Nitrogen

Na Sodium

Ni Nickel

O Oxygen

Pb Lead

S Sulphur

Sb Antimony

Sc Scandium

Se Selenium

Sn Tin

Sr Strontium

Ti Titanium

Tl Thallium

Zn Zinc

CO Carbon monoxide

NOx Nitrogen oxides

CO_2_ Carbon dioxide

SO_2_ Sulfur dioxide

HNO_3_ Nitric acid

H2O_2_ Hydrogen peroxide

CH_4_ Methane

H_2_O Water

NH_3_ Ammonia

O_2_r Percentage of reference oxygen content

O_2_m Percentage of oxygen measured

Cm Concentation of anlite measured
